# Adalimumab Treatment in Biologically Naïve Crohn's Disease: Relationship with Ectopic MUC5AC Expression and Endoscopic Improvement

**DOI:** 10.1155/2014/687257

**Published:** 2014-04-16

**Authors:** Tsutomu Mizoshita, Satoshi Tanida, Hironobu Tsukamoto, Keiji Ozeki, Takahito Katano, Hirotaka Nishiwaki, Masahide Ebi, Yoshinori Mori, Eiji Kubota, Hiromi Kataoka, Takeshi Kamiya, Takashi Joh

**Affiliations:** Department of Gastroenterology and Metabolism, Nagoya City University Graduate School of Medical Sciences, 1-Kawasumi, Mizuho-cho, Mizuho-ku, Nagoya 467-8601, Japan

## Abstract

*Background*. Adalimumab (ADA) is effective for patients with Crohn's disease (CD). However, there have been few reports on ADA therapy with respect to its relationship with pathologic findings and drug efficacy in biologically naïve CD cases. * Methods*. Fifteen patients with active biologically naïve CD were treated with ADA. We examined them clinically and pathologically with ectopic MUC5AC expression in the lesions before and after 12 and 52 weeks of ADA therapy, retrospectively. * Results*. Both mean CD activity index scores and serum C-reactive protein values were significantly lower after ADA therapy (*P* < 0.001). In the MUC5AC negative group, all cases exhibited clinical remission (CR) and endoscopic improvement at 52 weeks. In MUC5AC positive groups, loss of MUC5AC expression was detected in cases having CR and endoscopic improvement at 52 weeks, while remnant ectopic MUC5AC expression was observed in those exhibiting no endoscopic improvement and flare up after 52 weeks. * Conclusions*. ADA leads to CR and endoscopic improvement in biologically naïve CD cases. In addition, ectopic MUC5AC expression may be a predictive marker of flare up and endoscopic improvement in the intestines of CD patients.

## 1. Introduction

Anti-tumor necrosis factor- (TNF-) *α* inhibitors are important for treating Crohn's disease (CD) [1, 2]. In Japan, subcutaneous adalimumab (ADA, a fully human monoclonal antibody) and intravenous infliximab (IFX, a chimeric monoclonal antibody), both of which are TNF-*α* inhibitors, are approved for the treatment of CD [3, 4]. ADA induced and maintained clinical remission in patients with moderate to severe CD naïve to anti-TNF treatment, as shown in the CLASSIC I and CLASSIC II trials [5, 6]. ADA is also effective and well tolerated for inducing and maintaining clinical remission in Japanese patients with moderate to severe CD, particularly in cases naïve to anti-TNF treatment [7]. The CHARM Trial demonstrated that ADA both biweekly and weekly were significantly more effective than placebo in maintaining remission in moderate to severe CD among patients who responded to ADA [8]. In addition, subgroup analysis in the CHARM Trial showed increased remission rates through 3 years for ADA-treated patients with early CD, thus suggesting the importance of the top-down approach for the induction and maintenance of clinical remission [9]. However, CD cases undergoing ADA therapy are fewer than those undergoing IFX therapy, as ADA treatment of CD was approved about 10 years after IFX was permitted in Japan. In addition, with regard to ADA therapy for CD in Japan, there is little evidence regarding CD cases undergoing ADA treatment as the first TNF-*α* inhibitor (cases naïve to anti-TNF treatment), as most CD cases are treated with ADA after IFX therapy.

Some reports have demonstrated findings of ectopic gastric phenotypic expression, such as of MUC5AC, in inflammatory bowel diseases (IBDs) [10, 11] and in UC-associated dysplasia/neoplasms [12, 13]. The presence of MUC5AC correlated positively with inflammatory activity in UC [14]. We have previously shown that loss of ectopic MUC5AC expression is important for pathologic remission in the colon of UC patients [15]. With regard to ectopic gastric phenotypic expression in CD, gastric mucins (MUC5AC and MUC6) may have a role in epithelial wound healing after mucosal injury [10]. In the ulcer-associated cell lineage (UACL), expression of mucous cells with a foveolar structure showed immunoreactivity to MUC5AC, while mucous cells with a glandular structure showed immunoreactivity to MUC6, and expression of MUC2 was decreased, thus suggesting that UACL showed histological differentiation simulating gastric mucosa [16]. However, there are few reports on ectopic MUC5AC expression with respect to its relationship with endoscopic and clinicopathologic findings in CD cases.

In the present study, we therefore analyzed the expression of ectopic MUC5AC in the mucous cells of the large and small intestines in patients with CD undergoing ADA treatment as the first TNF-*α* inhibitor (cases naïve to anti-TNF treatment) before and at 12 and 52 weeks after the start of ADA therapy, using C-reactive protein (CRP), the CD activity index (CDAI), and CD endoscopic index of severity (CDEIS) scores as a measure of disease activity, retrospectively.

## 2. Patients and Methods

### 2.1. Patients and ADA Treatment

Between December 2010 and December 2012, 15 consecutive active CD patients (CDAI ≥150) naïve to anti-TNF treatment were administered subcutaneous ADA at Nagoya City University Hospital, after informed consent was obtained. Before the start of ADA, infectious enteritis, such as that caused by bacteria and cytomegalovirus,was ruled out by stool cultures,* Clostridium difficile* toxin testing, and pathological analysis of lesions.

According to the Japanese protocol, the patient received 160 mg of ADA by subcutaneous administration at week 0 and 80 mg at week 2 and subsequent subcutaneous administrations of 40 mg were given as a maintenance dose every other week thereafter.

### 2.2. Symptoms and Laboratory Assessment

Disease activity before and after subcutaneous ADA therapy was measured using the CD activity index (CDAI) score [5]. Response was defined as a reduction of ≥70 points (70-point response) or ≥100 points (100-point response) from week 0 in the CDAI score, and remission was defined as a CDAI score <150 points [5, 17]. We evaluated the CDAI score before and after 12 and 52 weeks of ADA administration in 15 patients having the ADA treatment as the first TNF-*α* inhibitor.

C-reactive protein (CRP) in particular was reported to correlate with disease activity [18]. We therefore evaluated serum levels of CRP (normal range ≤0.30 mg/dL) before and after 12 and 52 weeks of ADA administration in 15 patients having the ADA treatment as the first TNF-*α* inhibitor.

### 2.3. Endoscopic Assessment

Colonoscopy or double balloon endoscopy (DBE) was performed before and after 12 and 52 weeks of ADA administration. The colonoscopy was performed in all cases before the ADA administration. Regarding the evaluation of small intestine, the DBE was also performed when the lesions were detected by the small bowel series or computed tomography (CT) before the ADA administration. Endoscopic assessment of the lesions was evaluated according to CDEIS: nonactivity, CDEIS ≤3; mild active stage,  3≤ CDEIS < 9; moderate active stage, 9≤ CDEIS <12; and severe active stage, CDEIS ≥12 [19, 20].

### 2.4. Immunohistochemistry

In 15 CD patients undergoing ADA treatment as the first TNF-*α* inhibitor, biopsies from the inflamed mucosa in the small and large intestines were obtained before (*n* = 15) and after 12 (*n* = 14) and 52 (*n* = 14) weeks of ADA administration to evaluate histology and MUC5AC expression when endoscopy was performed. MUC5AC expression is generally detected in the cytoplasm of mucous cells of the stomach, while no MUC5AC expression is observed in the normal intestine. Immunohistochemical staining in the biopsy samples from the intestines of the CD patients receiving ADA administration was carried out with the following monoclonal antibody: MUC5AC (CLH2, 1 : 500; Novocastra Laboratories, Newcastle, UK). The precise procedures for immunohistochemical techniques were as described previously [15, 21]. Briefly, 4 *μ*m consecutive sections were deparaffinized and hydrated through a graded series of ethanol. After inhibition of endogenous peroxidase activity by immersion in 3% H_2_O_2_ methanol solution, sections were incubated with primary antibody, washed thoroughly in phosphate-buffered saline (PBS), and then incubated with biotinylated secondary antibody followed by the avidin-biotinylated horseradish peroxidase complex (Vectastain Elite ABC kit; Vector Laboratories, Burlingame, CA). Finally, immune complexes were visualized by incubation with 0.01% H_2_O_2_ and 0.05% 3,3′-diaminobenzidine tetrachloride (DAB). Nuclear counterstaining was accomplished with Mayer's hematoxylin.

Two independent investigators (Tsutomu Mizoshita and Hironobu Tsukamoto) judged the histology and immunohistochemical staining of MUC5AC in the cytoplasm of mucous cells of the colon, as described previously [15, 22].

### 2.5. Statistical Analyses

With regard to statistical analyses before and after ADA treatment, Wilcoxon* t*-test was applied to establish the significance of differences in the CDAI and CRP. *P* values of <0.05 were considered to be statistically significant.

## 3. Results

### 3.1. Patient Characteristics

Baseline characteristics of the 15 patients receiving subcutaneous ADA therapy are shown in Table 1. All patients received more than one year of ADA therapy. The male/female ratio was 10/5, and the median ages at diagnosis and start of therapy were 32.1 years (19–52 years) and 37.2 years (20–64 years) (median (range)), respectively. Median disease duration was 5.7 years (0.1–21 years) (median (range)). The 15 cases were divided into 3 L1, 7 L2, and 5 L3 types, according to the Montreal classification for CD. Six cases had perianal disease and 3 cases underwent previous surgical resection. Regarding the surgical resection, 2 cases had the ileocecal resection and 1 case had the ileocecal resection and the resection of the stricture in the small intestine twice. Regarding concomitant medication, 5 patients received prednisolone, 12 received 5-aminosalicylates, 4 received immunosuppressants (azathioprine, AZA), 5 received granulocyte and monocyte adsorptive (GMA) therapies, 4 received enteral nutrition, and none of them had previous use of IFX or biologic drugs (Table 1). None of the patients had serious adverse events requiring termination of ADA therapy. One case had hypertension, which was controlled by medication.

### 3.2. CDAI Score and CRP

**Figure 1:**
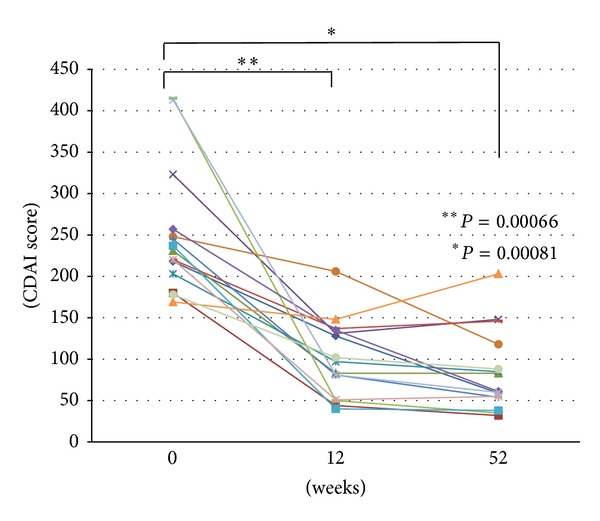
Crohn's disease activity index (CDAI) scores before and at 12 and 52 weeks after the start of adalimumab (ADA) therapy.

Mean CDAI score was significantly reduced from 250 ± 20 (average ± SE) at the start of subcutaneous ADA therapy to 101 ± 12 at week 12 in 15 CD patients naïve to anti-TNF treatment (*P* = 0.00066; Figure 1). Thirteen (86.7%) and 10 (66.7%) CD cases achieved to be 70-point and 100-point responses at week 12 after ADA therapy started. Fourteen (93.3%) patients achieved to be in clinical remission at week 12 after ADA therapy started. At week 52, mean CDAI score was significantly reduced from 250 ± 20 (average ± SE) at the start of the subcutaneous ADA therapy to 84 ± 13 (*P* = 0.00081, Figure 1). Fourteen (93.3%) and 12 (80.0%) CD cases achieved to be 70-point and 100-point responses at week 52 after ADA therapy started. Fourteen (93.3%) patients achieved to be in clinical remission at week 52 after ADA therapy started. In 14 cases (93.3%), clinical remission (CDAI score < 150 points) was maintained at week 52 by subcutaneous ADA therapy. As a result, inflammation flared up in a single case despite ADA therapy, suggesting a CDAI score ≥150 points at week 52 (Figure 1).

**Figure 2:**
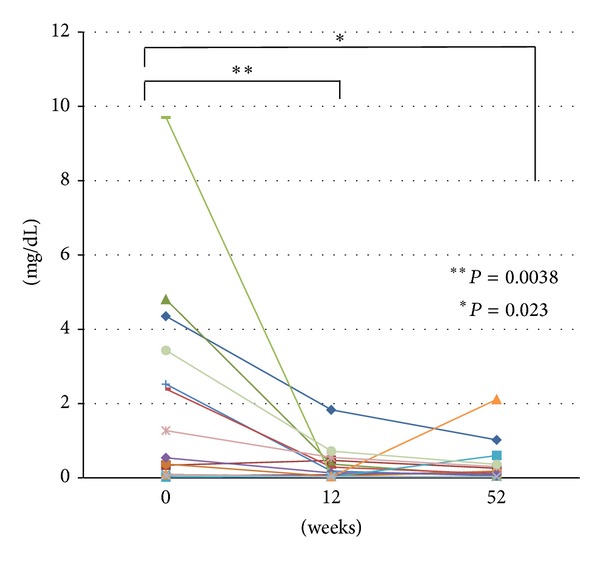
Serum levels of C-reactive protein (CRP) before and at 12 and 52 weeks after the start of adalimumab (ADA) therapy.

Mean CRP (normal range ≤0.30 mg/dL) was significantly reduced from 2.00 ± 0.70 (average ± SE) at the start of subcutaneous ADA therapy to 0.32 ± 0.12 at week 12 in 15 CD patients naïve to anti-TNF treatment (*P* = 0.0038, Figure 2). Mean value CRP was also significantly reduced from 2.00 ± 0.70 (average ± SE) at the start of the subcutaneous ADA therapy to 0.36 ± 0.14 at week 52, thus suggesting that ADA therapy inhibits the flare-up in CD cases (*P* = 0.023, Figure 2).

### 3.3. Relationships between MUC5AC and CD Patient Condition

**Figure 3:**
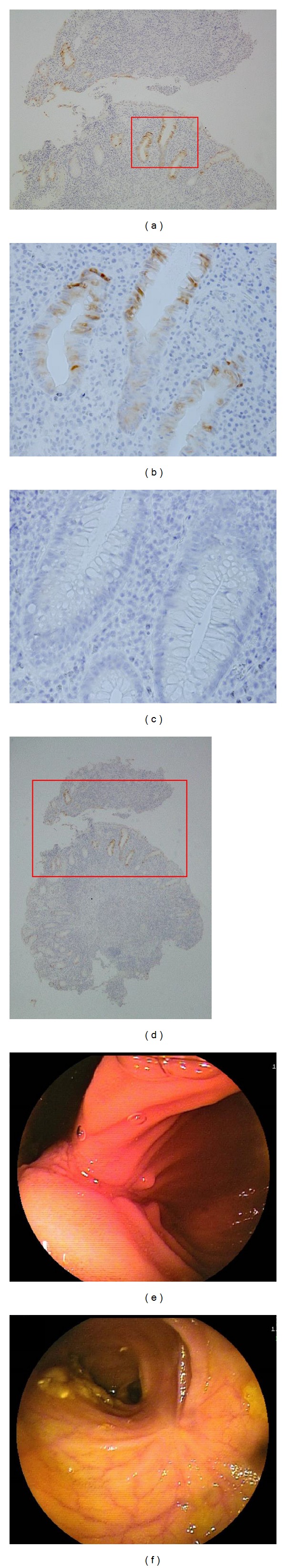
Ectopic MUC5AC expression is often positive in the cytoplasm of the mucous cells of the intestine in active CD (a, b, and d). Loss of MUC5AC expression is produced by adalimumab (ADA) in patients with remission stage (c). (a) ×100; (b) and (c) ×400; (d) ×40. Deep longitudinal ulceration was detected in the ileum by double balloon endoscopy (DBE) before ADA therapy (e) and the lesion showed mucosal healing after ADA therapy (f).

The relationships between MUC5AC expression, CDAI and CRP are summarized in Table 2. In the inflammatory mucosa of the small and large intestines, ectopic MUC5AC expression was observed in the upper part of the glandular duct and sometimes detected in the normal mucosa beside the inflamed one. Ectopic MUC5AC expression in the mucous cells of the small and large intestines was judged to be positive in 60.0% (9/15), 35.7% (5/14), and 21.4% (3/14) of CD patients before and after 12 and 52 weeks of ADA administration (Figures 3(a), 3(b), and 3(d)). In the MUC5AC-positive group (CD-ADA-1~9), 5 cases showed a loss of ectopic MUC5AC expression after 52 weeks of ADA therapy, thus suggesting clinical remission (CDAI < 150) (Figure 3(c)). However, the ectopic MUC5AC expression was detected in 3 CD cases at 52 weeks, and their condition became worse. In the 3 CD cases having ectopic MUC5AC expression, increases in CRP and CDAI (CDAI ≥150) were observed as signs of flare-up in 2 patients, while the remaining patient developed anal fistula, for which the Seton technique was performed surgically (Table 2). In the 3 CD cases with flare-up, we added AZA in 1 case, and we switched ADA to IFX as the second TNF-*α* inhibitor in 2 cases.

In the MUC5AC-negative group (CD-ADA-10~15), ectopic MUC5AC expression was not detected before or after 12 and 52 weeks of ADA administration. All 6 of these cases were judged to be in clinical remission (CDAI < 150) at 52 weeks.

The ectopic MUC5AC expression could not be evaluated in 1 case of MUC5AC-negative group after 12 weeks (CD-ADA-14) and 1 case of MUC5AC-positive group after 52 weeks (CD-ADA-5), since no endoscopic assessment was performed (Table 2).

Regarding the CDAI scores and CRP, we made the comparisons between MUC5AC-positive and MUC5AC-negative groups based on their baseline expression of MUC5AC before and after 12 and 52 weeks of ADA administration, but there were no statistical differences between two groups (data not shown).

### 3.4. CDEIS

CDEIS scores are summarized in Table 2. CDEIS scores at 12 and 52 weeks were reduced in 71.4% (10/14) and 78.6% (11/14) of patients, as compared to the scores before ADA therapy (Figures 3(e) and 3(f)). At 12 and 52 weeks, 3 and 4 cases, respectively, showed endoscopic mucosal healing. In the MUC5AC-negative group (CD-ADA-10~15), CDEIS scores at 12 and 52 weeks were lower in all cases, as compared to those before ADA therapy. In the MUC5AC-positive group (CD-ADA-1~9), CDEIS scores at 12 and 52 weeks decreased to 55.6% (5/9) and 62.5% (5/8), as compared to scores before ADA administration. In 3 CD cases in the MUC5AC-positive group with no endoscopic improvement and flare-up after 52 weeks, CDEIS exhibited moderate (1 case) or severe (2 cases) active stages, and all cases had ectopic MUC5AC expression in the intestines at 52 weeks (Table 2).

## 4. Discussion

The results of the present study show that loss/reduction of ectopic MUC5AC cytoplasmic expression in the mucous cells of the intestines is strongly associated with clinical remission in patients with CD. In the MUC5AC-negative group (*n* = 6), no ectopic MUC5AC expression was detected before or after 12 and 52 weeks of ADA administration. All 6 cases were judged to be in clinical remission at 52 weeks. In the MUC5AC-positive group (*n* = 9), 5 cases showed loss of ectopic MUC5AC expression after 52 weeks of ADA, thus suggesting clinical remission. Gel-forming mucins (particularly MUC5AC and MUC6) may play a role in epithelial wound healing after mucosal injury in inflammatory bowel disease, in addition to providing mucosal protection [10]. MUC5AC and TFF1 expression in goblet cells is common in inflammatory bowel disease and other inflammatory conditions of the colon, suggesting that these changes represent a nonspecific repair function of the colon cells to compensate for damage to barrier function [11]. With regard to the relationship between ulcerative colitis and ectopic MUC5AC expression in the mucous cells of the large intestines, patients with UC had levels above the threshold, and their mucosae were strongly labeled with anti-M1/MUC5AC antibody by immunohistochemistry [23]. The presence of MUC5AC correlated positively with inflammatory activity in UC [14]. Expression of gastric differentiation markers is potentially useful for detection of UC-associated dysplasia, suggesting that expression of gastric phenotype in the colon is important for UC-associated colorectal carcinogenesis [13]. Thus, gastric-type mucins may be useful in the differential diagnosis between UC-associated neoplasms and sporadic neoplasms [12]. We recently reported that loss of ectopic MUC5AC expression may be important for pathologic remission in the colon in UC patients [15]. In the present MUC5AC-positive group (*n* = 9), ectopic MUC5AC expression was detected in 3 cases at 52 weeks and their condition became worse, as was observed with ectopic MUC5AC expression in UC cases. Taken together with these previous reports, the present data suggest that loss of ectopic MUC5AC expression is important in achieving clinical remission status, and the remnant of ectopic MUC5AC is a sign of flare-up in CD patients. However, the present study is the retrospective analysis of small population. In addition, CD is a transmural disease, while MUC5AC is expressed in the mucous cells, suggesting the suspicious point that such an epithelial marker can be reliable in evaluating clinical remission in CD. Further studies may be needed to clarify whether reduction of ectopic MUC5AC expression affects positively remission status or not in the CD patients.

Regarding the mechanism of the regulation of MUC5AC expression, several reports have shown that TNF-*α* is a stimulant for secretion and gene expression of MUC5AC mucin in normal human airway epithelial cells [24–26]. Prunetin [27] and wogonin [28] are associated with the inhibition of MUC5AC mucin by the inhibition of the NF-*κ*B signaling pathway. In the stomach cancers and atrophic gastritis with* Helicobacter pylori* infection, it has been reported that SOX2 as a SRY-related HMG box protein and AT motif-binding factor 1 (ATBF-1) are associated with the regulation of MUC5AC expression [29–31]. Further studies also may be needed to clarify the regulation of MUC5AC expression by TNF-*α*, the above-mentioned factors, or other ones.

In the present CD cases naïve to anti-TNF treatment, 13 (86.7%) and 10 (66.7%) CD cases were judged to be 70-point and 100-point responses at week 12 after ADA therapy started. Fourteen (93.3%) patients were judged to be in clinical remission at week 12 after ADA therapy started. ADA induced clinical remission in patients with moderate to severe CD naïve to anti-TNF treatment, as shown by the CLASSIC I trial [5]. Fourteen (93.3%) and 12 (80.0%) CD cases were judged to be 70-point and 100-point responses at week 52 after ADA therapy started. Fourteen (93.3%) patients were judged to be in clinical remission at week 52. ADA maintained clinical remission in patients with moderate to severe CD naïve to anti-TNF treatment, as shown by the CLASSIC II trial [6]. In Japanese patients with moderate to severe CD, ADA is also effective and well tolerated for inducing and maintaining clinical remission, especially in cases naïve to anti-TNF treatment [7]. In addition, the median disease duration was 5.7 years (0.1–21 years) (median (range)) among the present CD cases, which is a relatively short disease duration. Subgroup analysis of the CHARM Trial showed increased remission rates through 3 years for ADA-treated patients with early CD, thus suggesting the importance of a top-down approach to the induction and maintenance of clinical remission [9]. These reports and the present data suggest that ADA therapy is effective for the induction and maintenance of clinical remission in CD patients naïve to anti-TNF treatment, particularly in early CD cases.

Mucosal healing is an increasingly important therapeutic goal in the treatment of patients with CD [20, 32, 33]. However, it is difficult to achieve endoscopic mucosal healing, as compared to clinical remission. Following induction therapy with ADA, CD patients who continue to receive ADA are more likely to achieve mucosal healing than those given placebo [20]. In the EXTEND Trial, 27% and 24% of CD patients receiving ADA had mucosal healing at 12 and 52 weeks [20]. Among our cases, 3 (21.4%) and 4 (30.8%) cases had endoscopic mucosal healing at 12 and 52 weeks. In the MUC5AC-negative group, CDEIS scores at 52 weeks were reduced in all cases, while those at 52 weeks were reduced in 62.5% (5/8) of the MUC5AC-positive group exhibiting the loss of ectopic MUC5AC expression in the intestines (Table 2). We believe that ADA therapy is more effective in CD cases who exhibit no ectopic MUC5AC expression before the therapy or loss of ectopic MUC5AC expression through the therapy from the viewpoint of the endoscopic improvement. In the 3 CD cases in the MUC5AC-positive group having no endoscopic improvement and flare-up after 52 weeks, CDEIS exhibited moderate (1 case) or severe (2 cases) active stages, and all 3 cases had ectopic MUC5AC expression in the intestines at 52 weeks, supporting our hypothesis that ectopic MUC5AC expression is a predictive marker of the endoscopic improvement in the intestines of patients with CD.

In conclusion, ADA brings clinical remission and endoscopic improvement to CD patients naïve to anti-TNF treatment. In addition, ectopic MUC5AC expression may be a predictive marker of flare-up and endoscopic improvement in the intestines of patients with CD.

## Figures and Tables

**Table 1:** Patients' Baseline Characteristics (*n* = 15).

Sex (Male/Female)	10/5
Age at diagnosis	32.1 (19–52)
[median (range)] (years)	
Age at start of the therapy	37.2 (20–64)
[median (range)] (years)	
Disease duration	5.7 (0.1–21)
[median (range)] (years)	
Extent of disease	
L1 (%)	3 (20.0%)
L2 (%)	7 (46.7%)
L3 (%)	5 (33.3%)
Perianal disease (%)	6 (40.0%)
Previous surgical resection (%)	3 (20.0%)
Concomitant medication	
Predonisolone	5
5-Aminosalicylates	12
Immunosuppresants (AZA)	4
GMA	5
Enteral nutrition	4
Anti-TNF^a^	0

AZA: azathioprine; GMA: granulocyte and monocyte adsorptive apheresis; L1: Ileum; L2: Colon; L3: Ileocolon.

^a^Previous use of infliximab or biologic.

**Table 2:** The relation between MUC5AC immunostaining, CDAI, CRP and CDEIS in the CD patients having the ADA treatment as the first TNF-*α* inhibitor (*n* = 15).

	CDAI			CRP			MUC5AC			CDEIS			Clinical course after 52 weeks
	0 w	12 w	52 w	0 w	12 w	52 w	0 w	12 w	52 w	0 w	12 w	52 w	
CD-ADA-1	Moderate	Remission	Remission	+	−	−	2	2	2	3+	3+	3+	The seton operation was performed, since anal fistula worsened.
CD-ADA-2	Mild	Remission	Mild	−	−	+	1	0	2	1+	1+	3+	Flare up (CRP = 2.11↑)
CD-ADA-3	Moderate	Remission	Remission	−	−	+	1	1	2	2+	1+	2+	Flare up (CRP = 6.09↑) after 78 weeks
CD-ADA-4	Moderate	Mild	Remission	+	−	−	2	2	0	2+	2+	1+	Maintenance of clinical remission
CD-ADA-5	Mild	Remission	Remission	+	+	+	2	2	—	3+	1+	—	Maintenance of clinical remission
CD-ADA-6	Moderate	Remission	Remission	−	−	−	2	1	0	3+	1+	MH	Maintenance of clinical remission
CD-ADA-7	Moderate	Remission	Remission	+	+	−	2	0	0	3+	3+	1+	Maintenance of clinical remission
CD-ADA-8	Moderate	Remission	Remission	+	+	−	1	0	0	3+	MH	MH	Maintenance of clinical remission
CD-ADA-9	Moderate	Remission	Remission	+	−	−	1	0	0	3+	1+	1+	Maintenance of clinical remission
CD-ADA-10	Mild	Remission	Remission	+	+	−	0	0	0	3+	MH	2+	Maintenance of clinical remission
CD-ADA-11	Mild	Remission	Remission	+	+	+	0	0	0	3+	1+	1+	Maintenance of clinical remission (CRP was judged to be negative after 78 weeks.)
CD-ADA-12	Moderate	Remission	Remission	−	−	−	0	0	0	3+	1+	2+	Maintenance of clinical remission
CD-ADA-13	Mild	Remission	Remission	−	−	−	0	0	0	2+	1+	1+	Maintenance of clinical remission
CD-ADA-14	Moderate	Remission	Remission	+	−	−	0	—	0	3+	—	MH	Maintenance of clinical remission
CD-ADA-15	Moderate	Remission	Remission	+	−	−	0	0	0	3+	MH	MH	Maintenance of clinical remission

CDAI: Remission: score < 150; Mild: 150 ≤ score < 220; Moderate: 220 ≤ score < 450; Severe: score ≥ 450.

CRP: −: ≤0.30 mg/dL; +: >0.30 mg/dL.

CDEIS: non-activity (mucosal healing: MH): CDEIS < 3; mild active stage (1+): 3 ≤ CDEIS < 9; moderate active stage (2+): 9 ≤ CDEIS < 12; severe active stage (3+): 12 ≤ CDEIS.
